# Acknowledgement to Reviewers of *Viruses* in 2013

**DOI:** 10.3390/v6031073

**Published:** 2014-03-05

**Authors:** Viruses Editorial Office

**Affiliations:** MDPI AG, Klybeckstrasse 64, CH-4057 Basel, Switzerland

The editors of *Viruses* would like to express their sincere gratitude to the following reviewers for assessing manuscripts in 2013:

Abbate, Isabella
Abdelwhab, E. M.
Abecassis, Michael
Abedon, Stephen
Abendroth, Allison
Abrahao, Jonatas
Accotto, Gian Paolo
Ahlm, Clas
Aiken, Christopher
Alexander, Plyusnin
Amarasinghe, Gaya K.
Amet, Tohti
Amuzie, Chidozie
Andreadis, Ted
Angelopoulou, Katerina
Aquaro, Stefano
Arbiza, Juan
Arbuckle, Jesse
Arhel, Nathalie J.
Ascencio-Ibáñez, José Trinidad
Aspinall, E. J.
Attoui, Houssam
Avdic, Selmir
Avsic-Zupanc, Tatjana
Azab, W. F.
Bagamian, Karoun
Baldanti, Fausto
Bannert, Norbert
Bao, J.
Barr, John N.
Barry, Peter
Bartosch, Birke
Battisti, C. De
Bausch, Dan
Beard, Brian C.
Beasley, David
Beaumelle, Bruno
Bejarano, Eduardo
Bell, Thomas
Benaroch, Philippe
Benavides, Julio
Benmohamed, Lbachir
Berges, B. K.
Berkson, Bm
Bernasconi, P.
Berriatua, E.
Bertoni, Giuseppe
Bharate, Sandip B.
Bisanzio, D.
Blacklaws, Barbara
Blair, Carol D.
Blondel, Danielle
Bloom, David C.
Bolling, B.
Bong-Kyun, Park
Bonning, Bryony C.
Bordon, Jose M.
Boscia, Donato
Botten, Jason
Bouamr, Fadila
Bouchard, Michael J.
Boyce, Walter
Boydston, Jeremy A.
Bradfute, Steven
Brandt, Curtis R.
Brault, Aaron
Briddon, Rob
Brierly, Ian
Brinton, Margo
Broder, Christopher
Brune, Wolfram
Brunotte, Linda
Buckling, Angus
Bukrinsky, Michael
Buller, Mark
Burger, Johan
Burkhalter, Matthias
Calisher, Charles
Cannon, Paula
Carbajo, Anibal
Cardin, Rhonda
Casas, Eduardo
Catoni, Marco
Chackerian, B.
Chandran, Kartik
Chao, Sheng-Hao
Chen, Benjamin
Chen, H.-L.
Chen, Jason
Chen, Mei - Ru Chen
Chen, Steve S.-l.
Chen, Wuguo
Chen, Zhi-Nan
Chen, Zhiwei
Cherepanov, Peter
Cho, Nam Hoon
Choi, Seung-Kook
Chuang, Ting-Wu
Cicin-Sain, Luka
Cimarelli, A
Clapham, Paul
Clark, Jason
Clem, Rollie
Clementi, Nicola
Cocquerel, Laurence
Cohen, Gary
Cohrs, Randy
Colberg-Poley, Anamaris M.
Compans, Richard W.
Constantine, Niel
Costa, Luciana
Darós, José Antonio
Dash, Srikanta
Datta, Siddhartha A.K.
De Andres, Damian
De Silva, Aravinda
Decaro, Nicola
Del Real, Gustavo
Detlef, Michel
Dewitte-Orr, Stephanie
Diaz-San Segundo, Fayna
Di Martino, Barbara
DiGiusto, David
Dirk, Lindemann
Diserio, Francesco
Dittmer, Dirk
Dobner, Thomas
Dombrovsky, Aviv
Donlin, Maureen
Drebot, Mike
Dropulić, Boro
Du, Zhiyou
Ebihara, Hideki
Ebina, Hirotaka
Elankumaran, Subbiah
Elder, John
Essbauer, Sandra
Evander, Magnus
Everett, Roger D.
Faaberg, Kay S.
Fair, Jeanne
Falzarano, Darryl
Farkas, Silvie
Fearns, Rachel
Fehse, Boris
Feldmann, Heinz
Feng, Zongdi
Fernandes, Patricia
Finke, Stefan
Fischer, Matthias
Flemington, Erik K.
Flores, Ricardo
Fontoura, Beatriz M.
Fortunato, Elizabeth
Fossen, Torgils
Fredericksen, Brenda L.
Freed, Eric
Fuchs, Marc
Gagnon, Carl A.
Gallicano, G. Ian
Gallitelli, Donato
Gao, Feng
García, M.
Garten, Wolfgang
Gasque, Philippe
Geng, Xin
Georgel, Philippe
Gerard, Norma
Gessain, Antoine
Gewurz, Ben
Ghanim, Murad
Giacca, Mauro
Gilbert, M.
Gilden, Donald H.
Glineur, Stephanie
Goff, Steve
Golde, William T.
Goldmacher, Victor
Golisz, Anna
Goodbourn, Stephen
Gottikh, M. B.
Gowen, Brian
Gray, Wayne
Grolla, Allen
Grose, Chuck
Groseth, Allison
Guo, Ju-tao
Gustin, Kurt
Hahn, Thomas von
Hall, R.A.
Hampson, Ian N.
Han, Guan-zhu
Harit, Dimple
Harper, David
Harrich, David
Hausmann, Juergen
Haverkos, Harry W.
Hayman, David
He, Nongyue
Hefferon, Kathleen Laura
Hellen, Christopher U. T.
Hepojoki, J.
Heyman, Paul
Hill, James M.
Hirsch, Alec J.
Hirsch, Vanessa
Hjelle, Brian
Hoeben, Rob C.
Hoelz, André
Hoenen, Thomas
Hofmann, Janin
Hoppe-Seyler, Felix
Hotzel, Isidro
Hrincius, Eike
Hsia, Victor
Hsu, Wei-Li
Hu, Nan
Hu, Xiufang
Huang, F.
Hubalek, Zdenek
Hummel, Mary
Hunter, Nora
Huthoff, Hendrik
Ilyas, Muhammad
Isogai, Masamichi
Ito, Toshihiro
Izumiya, Yoshi
J. Kuhn, Richard
Jacobs, N.
Jangra, Rohit
Jenson, Vicki
Jenson, Victoria
Jiro, Arikawa
Johnson, Nicholas
Johnson, Welkin
Jolly, Clare
Jones-Engel, Lisa
Jonsson, Colleen
Joshi, Anjali
Kadowaki, Tatsuhiko
Kalejta, Robert
Kamil, Jeremy P.
Kariwa, Hiroaki
Katz, Richard
Kay, Michael S.
Kearney, Mary
Kerry, Julie
Khan, Mazhar
Kim, Jae-Hong
Kitajima, M.
Klein, Sabra
Klempa, Boris
Klenk, Hans-Dieter
Klingström, Jonas
Klöhn, Peter-Christian
Klupp, Barbara
Kniel, Kalmia E.
Kobinger, Gary
Koerkel, Maria
Kohara, Michinori
Kohl, Alain
Koike, Satoshi
Komar, Nicholas
Kousoulas, Konstantin G.
Koyanagi, Yoshio
Kramer, Vicki
Krautkramer, Ellen
Krishnan, Manoj N
Kristenson, Krister
Kumar, Ajit
Lai, Michael M.C.
Laimins, Laimonis A.
Laine, Outi
Lambrecht, Bénédicte
Landry, Nathalie
Lawler, James
Lawson, Victoria
Lecollinet, Sylvie
Lee, Jin-ching
Lee, Sunhee
Leggatt, G.
Leis, Jonathan
Lemos, Elba R. S.
Lemmerman, Niels
Leroux, Caroline
Levy, Laura
Li, Fang
Li, Feng
Lieberman, Paul M.
Lim, Jean
Lin, Junyan
Lindenbach, Brett
Ling, Anna PK
Linial, Maxine
Lippé, Roger
Liu, Qinfang
Liu, Shan-Lu
Liu, Wanhong
Lobigs, Mario
Lochelt, Martin
Löchelt, Martin
Lowenberger, Carl
Lozach, Pierre-Yves
Lu, Jie
Lucifora, Julie
Lundstrom, Kenneth
Mackay, Ian M
Mackow, Erich
Malathi, V. G.
Mann, Brian
Mansour, Mena
Marfin, Anthony
Margulies, David
Martin, M.
Martínez-Salas, Encarnación
Mateu, Enric
Matho, Michael
Matsuzaki, Shigenobu
Maury, Wendy
Mawassi, Munir
Mcbride, Alison
Mcgavern, Dorian
Mckenzie, Deb
McMenamy, Michael J.
McVoy, Michael
Medalia, Ohad
Melero, José A.
Menendez-Arias, Luis
Mergia, Ayalew
Messerle, Martin
Metwally, Ashraf
Meyer, Justin
Meyers, Craig
Milligan, Gregg
Mills, James
Mitsuma, Sheila
Miyoshi-Akiyama, T.
Mocarski, Edward
Montgomery, Ruth R
Moore, Martin
Morales, R.
Morello, Chris
Morgan, Iain M.
Morita, Eiji
Morrey, John
Müller, Barbara
Murphy, Eain
Myles, Kevin M.
Nagata, Kyosuke
Nakanishi, Akira
Nasci, Roger
Navas-Castillo, Jesus
Netland, Jason
Neumann, Donna M.
Neumann, Peter
Nevels, Michael
Nie, Xianzhou
Niedrig, Matthias
Noris, Emanuela
O'Keefe, Barry R.
Ohno, Mutsuhito
Olson, Kenneth E.
Ooka, Tadamasa
Oren, Kobiler
Ott, M
Padula, Paula
Paeshuyse, Jan
Pagano, Joseph S.
Paintsil, Elijah
Palese, Peter
Palmenberg, Ann
Panganiban, Nito
Pankaj, Kumar
Paprotka, Tobias
Parent, Leslie
Pari, Gregory
Parish, Joanna
Parolin, Cristina
Patil, Basavaprabhu
Pavlovic, Jovan
Peeples, Mark
Peng, Zong Gen
Peterschmitt, Michel
Petersen, Lyle R.
Piccone, Maria E.
Pietschmann, Thomas
Pijlman, Groben
Piles, Vicente Medina
Pintó, Rosa M.
Poehlmann, Stefan
Poeschla, Eric
Pooggin, Mikhail M.
Prescott, Joseph
Prichard, Mark N.
Puranaveja, Suphasawatt
Qadri, Ishtiaq
Qiao, Wentao
Raab-Traub, Nancy
Rai, Devendra
Rappocciolo, Giovanna
Rawlinson, William D
Ray, Stuart
Regamey, Nicolas
Rein, Alan
Reina, Ramses
Renault, T.
Reperant, Leslie
Reva, Oleg
Rezende, Jorge
Rice, Andrew
Rice, Stephen A.
Richman, Douglas
Rifo, Ricardo Soto
Rimstad, Espen
Rivera-Toledo, Evelyn
Rodríguez, Dolores
Rohemail, Changhyun
Rollier, Christine S
Romalde, Jesús López
Rosati, Sergio
Rosenberg, Helene
Rosenthal, Ken
Roth, Justin
Roy, Chad
Roy, Polly
Roy, Somnath
Ruprecht, Klemens
Rusvai, Miklos
Saib, Ali
Saldarelli, Pasquale
Sample, Clare
Sandmeyer, Susan
Sarid, Ronit
Sather, Noah
Schang, Luis
Schirrman, Thomas
Schlee, Martin
Schleiss, Mark
Schmaljohn, Connie
Schneider, William
Schols, Dominique
Schröder, Martina
Schwemmle, Martin
Scott, Rona
Sean, Polen
Séron, Karin
Serracca, Laura
Seto, Donald
Shapiro, Lawrence
Shi, Pei-Yong
Shin, Injae
Shinya, Kyoko
Siddiqui, Aleem
Sijts, Alice
Sinclair, Allison J.
Sinclair, John
Slagle, Betty
Slavinski, Sally
Smit, Jolanda
Smithgall, Thomas E.
Snyder, Chris
Song, Jin-Won
Sourvinos, George
Spann, Kirsten
Spatz, Steve
Spengler, Jessica
Spiropoulou, Christina
Stadler, Michael
Staeheli, Peter
Stamminger, Thomas
Stanton, Richard
Steensels, Mieke
Steinmann, Joerg
Stoye, Jonathan
Strebel, Klaus
Striker, Robert
Strong, James
Strong, Jim
Sullivan, Christopher
Suo, Siqingaowa
Suthar, Mehul
Suzuki, Nobohiro
Swamy, Manjunath N.
Switzer, William
Tanaka, Yasuhito
Tandon, Ritesh
Taylor, Shannon
Teng, Yong
Tenney, Daniel J.
Terajima, Masanori
Terzieva, Vesilava
Thomas, Christopher
Thomason, Lynn
Thompson, Jeremy R.
Thorley, Bruce
Thormar, Halldor
Thorne, Claire
Tiwari, Vaibhav
Tolari, Francesco
Torimiro, Judith
Torres-Perez, Fernando
Torsteinsdottir, Sigurbjorg
Trobridge, Grant
Ulbert, Sebastian
Ulrich, Rainer
Van Etten, James
Van Maarseveen, Noortje M
Varallyay, Eva
Varga, Steven
Vergara, Julia
Vlasova, Anastasia N.
Volkman, Loy E.
Volz, Asisa
von Laer, Dorothee
Wang, Qian
Wang, Qiuhong
Wang, Tina
Wang, Xiaowei
Watanabe, Toshiki
Watashi, Koichi
Webster, Robert
Weitzman, Matthew
Wilson, Stuart A
Worgall, Stefan
Wright, Jerry Mobley
Xing, S.
Xing, Sifei
Xu, Wenxing
Yanagihara, Richard
Yang, J.S.
Yang, Z.-Q.
Yeh, S.-d.
Yoshikawa, Nobuyuki
You, Jianxin
Zaia, John
Zamorano, Patricia
Zarlenga, Dante
Zhang, Gai-ping
Zhang, Jiuchun
Zhao, Yingming
Zhirnov, Oleg
Ziegelbauer, Joseph
Ziegelbauer, Joseph M.
Ziegler, Angelika

